# Tribological and Mechanical Applications of Liquid-Crystal-Polymer-Modified Carbon-Fiber-Reinforced Polyamide–Polyurethane Composites

**DOI:** 10.3390/polym15092033

**Published:** 2023-04-25

**Authors:** Zhen Zhou, Xiaoqing Wang, Xiaomeng Li, Chang Liu, Guoping Li, Yunjun Luo

**Affiliations:** Beijing Institute of Technology, School of Materials Science and Engineering, Beijing 100081, China; 3120201245@bit.edu.cn (Z.Z.);

**Keywords:** liquid crystal polymer, graphite, fiber-reinforced composites, tribological properties

## Abstract

An aromatic copolyester liquid crystal polymer (LCP) was introduced into carbon-fiber-reinforced polyamide–polyurethane (CF/PA-PU) composites through melt blending to improve the tribological properties of the composites. The effects of LCP on the mechanical, processing, and thermal properties of CF/PA-PU composites were compared to those of commonly-used graphite (Gr). The results showed that at 5 wt.% LCP content, the coefficient of friction (COF) was decreased by 16.06%, and the wear rate by 32.22% in the LCP/CF/PA-PU composite compared to the CF/PA-PU composite. Furthermore, using LCP instead of Gr showed significantly improved mechanical properties and reduced processing viscosity. The tensile strength of 5%LCP/CF/PA-PU composite could reach 99.08 MPa, while the equilibrium torque was reduced, being 26.85% higher and 18.37% lower than those of CF/PA-PU composite, respectively. The thermal stability of LCP/CF/PA-PU composites was also enhanced. The addition of 5 wt.% LCP to CF/PA-PU composite increased the initial decomposition temperature by 14.19% compared to CF/PA-PU. In sharp contrast, the addition of Gr increased equilibrium torque and actual processing temperature leading to processing difficulties and instability. This approach offers a novel strategy for tribological applications and tackles the problem of high viscosity in CF/PA-PU composites.

## 1. Introduction

Polyamide–polyurethane (PA-PU) block copolymers could improve the structural stability of polyamide, addressing its issues of low-notched impact strength and poor toughness [1,2,3]. While PA-PU copolymers are expected to replace metal and ceramic materials in gears, bearings, and other critical components, they still face challenges due to the low wear resistance under sliding conditions, which makes them prone to failure in mechanical parts [4,5]. Therefore, the enhancement of friction and wear resistance of the PA-PU copolymers is essential for their practical applications.

The introduction of carbon fiber (CF) into polymer matrices is a widely used approach to improve the mechanical and wear properties [6,7,8]. While the approach is effective and convenient, excessive amounts of CF can lead to high viscosity during processing, resulting in several issues such as poor dispersion, incomplete interfacial compatibility, and unreliable product stability. These issues ultimately hamper the overall performance [9,10]. Li et al. [8] found that internal defects in CF/PA6 composites were responsible for decreased tensile strength. The concentration of 35 wt.% CF hindered the flow of the polymer matrix, causing increased porosity and inadequate impregnation. Another study revealed that the addition of 30 wt.% CF to the composite resulted in inferior dispersion and greater stress concentrations in the matrix, which promoted detachment of fillers and degraded the tribological performance of the CF/PA6 [11]. Many attempts have focused on fiber modification to improve the interfacial interaction between carbon fiber and polymer matrix [12,13,14]. However, the enhancement of CF modifications is limited by the potential impacts of compromising the strength of CF itself or complicating the preparation process.

Many studies have demonstrated that common fillers such as graphite (Gr) [15,16], MoS_2_ [17], wax [18], and PTFE [19] can be used economically and effectively as friction modifiers in composites. However, the fillers as described above tend to come at the expense of other properties deteriorating, which could negatively impact the practical production and application. For instance, when 10 wt.% Gr was added into the thermoplastic polyurethane (TPU) blend, a reduction in friction was achieved but with an adverse effect on the wear rate, while causing viscosity to increase 2.3 times and strength to decrease by 70% compared to the TPU blend [20]. Multi-component hybrid reinforcements have also been reported for improving tribological properties in polyamide or polyurethane composites [21,22,23]. Vikram et al. [21] discovered that incorporating 5% glass fiber with 1% graphite could decrease the coefficient of friction and specific wear rate of the composite while increasing the tensile strength and modulus up to 1.5 times compared to pure PA66.

In recent times, there has been a growing interest in the thermotropic liquid crystal polymer (LCP) as a self-reinforcing material, due to its ability to undergo in situ fibrillation under appropriate processing conditions [24]. Moreover, an aromatic copolyester LCP has been utilized in poly (vinylidene fluoride) (PVDF) composites to improve the tribological properties, taking advantage of the lubricating shear planes formed by the conjugated benzene rings of LCP [25]. Particularly, the high shear thinning and orientability properties of LCP could reduce the processing viscosity of composites [26,27,28]. Therefore, it is a potential strategy to utilize LCP via rational formulation to further improve the tribological properties of CF/PA-PU composites while optimizing the current state of high viscosity resulting from CF.

In this work, LCP, an aromatic copolyester mainly composed of 1,4-hydroxybenzoic acid (HBA) and 2,6-hydroxynaphthoic acid (HNA), was introduced into a CF/PA-PU composite system to improve the tribological properties through the synergistic effect of the functional filler and carbon fibers, while meeting higher demands for mechanical and processing properties. Meanwhile, the typical filler graphite was compared with LCP, and the tribological, tensile, processing, and thermal properties of both LCP/CF/PA-PU and Gr/CF/PA-PU composites were systematically investigated. Our work demonstrates that LCP is a novel and effective filler to improve the tribological properties of CF/PA-PU composites. Moreover, it has great potential to further improve the mechanical properties and overcome the processing difficulties associated with CF/PA-PU composites compared with the conventional rigid filler. This approach provides an alternative and promising strategy regarding the tribological applications of polymeric systems.

## 2. Materials and Methods

### 2.1. Materials

Commercially available polyamide–polyurethane (PA-PU) block copolymer pellets (trade name: TM05400, Mn = 28097, with a melt flow index of 12 g/10 min at 235 °C and 1.2 kg, and water absorption rate of 1.33%) were provided by Tianjin Aini Electromechanical Co., Ltd., China. Pitch-based short carbon fiber (trade name: G-16, with a diameter of 20 um, length of 3 mm, and bulk density of 55–70 g/L) was commercially obtained from Hunan Dongbang New Materials Technology Co., Ltd., China. Thermotropic liquid crystal polymer (trade name: Vectra^®^ V400 P, with a density of 1.40 g/cm^3^, water absorption rate of 0.04%, and recommended melt processing temperature of 225–235 °C) was purchased from Celanese Corporation, America. Graphite powers (99.95% metal basis, 800 mesh) was purchased from Macklin, China. Antioxidant 1010 (C_73_H_108_O_12_, pentaerythritol tetrakis (3,5-di-tert-butyl-4-hydroxyhydrocinnamate)) as primary antioxidant and Antioxidant 168 (C_42_H_63_O_3_P, tris(2,4-di-tert-butylphenyl) phosphite) as secondary antioxidant were purchased from Aladdin, China.

### 2.2. Preparation of PA-PU Copolymer Composites

**Scheme 1:**
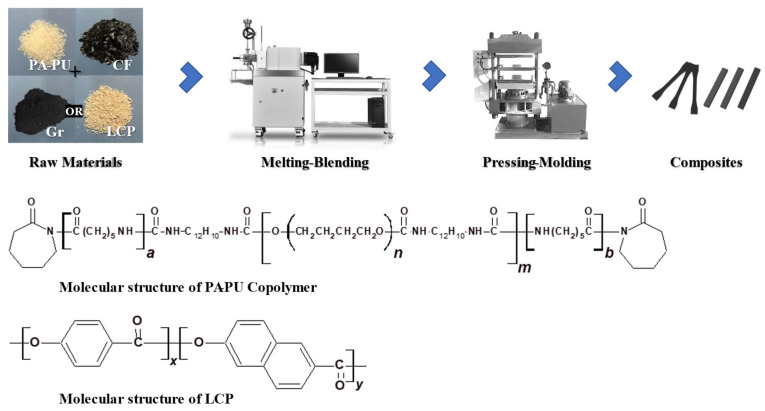
Illustration of the preparation of CF/PA-PU composites containing Gr or LCP through melt blending and press molding.

Before melt blending, all materials were dried at 70 °C for 12 h in a vacuum oven. According to a pre-designed formulation, CF/PA-PU composites with different contents of LCP or Gr were prepared by using a metering mixer (model 30/50EHT, Brabender Gmbh & Co. KG, Germany) at 240 °C with a rotation rate of 30 rpm for 10 min each. The composites were crushed and dried at 70 °C for 8 h to be ready for press molding. Standard tensile test specimens in dumbbell shape and standard friction and wear test specimens were press-molded by using a plate vulcanization machine (model DR-25, Yangzhou Derui Instrument Equipment Co., Ltd.) at a mold temperature of 230 °C and a mold pressure of 15 MPa. Finally, the specimens were annealed in a vacuum oven at 80 °C for 1 h to eliminate the residual stress generated during processing. The preparation process is shown schematically in Scheme 1. The detailed formulations of the CF/PA-PU composites are presented in Table 1.

### 2.3. Characterization

Tensile properties were tested using a universal tensile machine (Instron 5966, Illinois Tool Works Inc., Glenview, IL, USA), and a tensile speed of 20 mm·min^−1^. A minimum of five specimens were tested for each set of specimens and the average results were reported. The tribological test was carried out on a tribometer (TG-8200-P, Dongguan Maisheng Electronic Equipment Co., Ltd., Dongguan, China) at a constant normal sliding speed of 200 rpm and applied load values of 200 N without lubrication. The tests were carried out for 2 h, and three tribological tests were performed on each set of specimens. The coefficient of friction was monitored throughout the tests. After the tribological and tensile tests, scanning electron microscope (SEM) examinations of the wear and fracture surfaces were carried out on a Hitachi S4800 microscope, Hitachi, Ltd., Tokyo, Japan. The surface of each specimen was coated with gold for a detailed examination of morphological behavior. The processing properties were characterized using a torque rheometer (Model 30/50EHT, Brabender Gmbh & Co. KG, Duisburg, Germany), which can provide the changes in torque, temperature, and energy consumption during processing. Thermogravimetric analysis (TGA) was performed in an N_2_ atmosphere using a thermal gravimetric analyzer (DSC/TGA1, Mettler Toledo Company, Greifensee, Switzerland). The mass of each sample was 8–10 mg. Samples were ramped from room temperature to 600 °C at a heating rate of 10 K·min^−1^ while maintaining an N_2_ flow of 50 mL·min^−1^.

## 3. Results and Discussion

### 3.1. Tensile Properties

**Figure 1:**
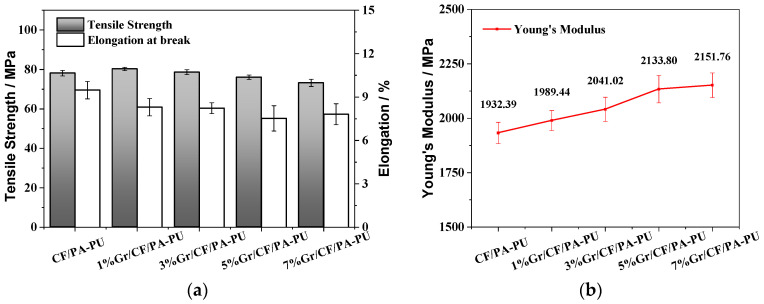
(**a**) Tensile strength, elongation at break, and (**b**) Young’s modulus of CF/PA-PU composites with different contents of Gr.

**Figure 2:**
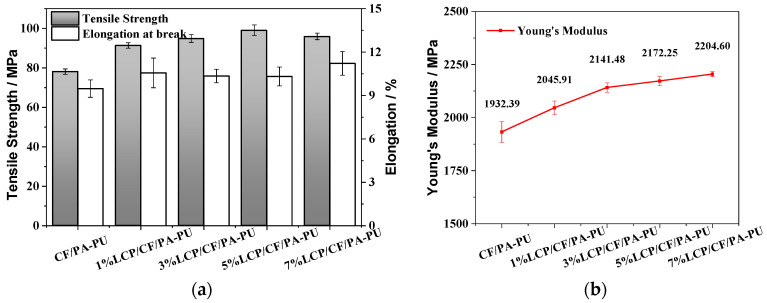
(**a**) Tensile strength, elongation at break, and (**b**) Young’s modulus of CF/PA-PU composites with different contents of LCP.

Mechanical properties are the primary consideration in the engineering application of composites, and excellent mechanical strength is a prerequisite for good friction and wear properties. Therefore, the effects of Gr and LCP on the tensile properties of CF/PA-PU composites were comparatively investigated. Figure 1 and Figure 2 show the tensile properties of CF/PA-PU composites with different contents of Gr and LCP, respectively, including tensile strength, elongation at break, and Young’s modulus.

**Figure 3:**
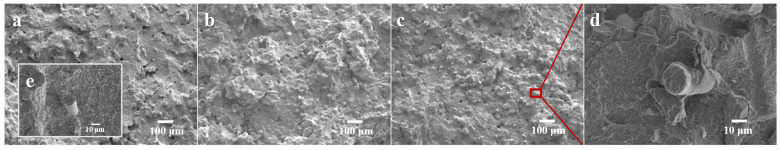
SEM images of the tensile fracture surfaces of the specimens: (**a**) CF/PA-PU, (**b**) 1%Gr/CF/PA-PU, and (**c**) 5%LCP/CF/PA-PU composites. The SEM images of (**d**,**e**) are taken at high magnification of (**c**) and (**a**).

Based on its strength and affinity for the polymer matrix, the right amount of Gr could reinforce the tensile properties of the CF/PA-PU composite. As shown in Figure 1a, the tensile strength of Gr/CF/PA-PU composites initially increases and then decreases as the amount of Gr is increased. The 1%Gr/CF/PA-PU has a maximum tensile strength of 80.26 MPa, which is 2.75% higher than that of the CF/PA-PU composite (78.11 MPa). In the CF/PA-PU composites, carbon fibers are used as a skeleton to carry the load. The surface effect of Gr is used to bind polymer molecular chains on the particle surface and form physical crosslinking between the chains, which serves to distribute the load uniformly [16]. Simultaneously, the uniform distribution of Gr within the matrix is beneficial for preventing crack propagation in the composites, as shown in Figure 3b, and thus the addition of 1 wt.% Gr plays a reinforcing role. However, the increased Gr content will inevitably lead to the inhomogeneity of distribution and incompatibility with polymers, which will cause defects in the composite and reduce the tensile strength [21]. Therefore, the tensile strength of Gr/CF/PA-PU composites decreases over the Gr content of 1%. Meanwhile, the elongation at break of Gr/CF/PA-PU composites is lower than that of CF/PA-PU composites, and the elongation at break decreases with an increase in Gr content. Different from the trend of tensile strength, as shown in Figure 1b, the Young’s modulus of the Gr/CF/PA-PU composite essentially increases as the Gr content increases. This means that with an increase in Gr content, the bond between the polymer molecular chain itself or with the filler strengthens, resulting in improved resistance to deformation within the elastic range.

Compared to Gr, the thermotropic liquid crystal polymers (LCPs), as self-reinforced materials, utilize their unique ordered orientation structure and flow properties [27]. As shown in Figure 2a, the tensile strength of LCP/CF/PA-PU composites shows a trend of initially increasing and then decreasing as the LCP content increases, which is like that presented in Gr/CF/PA-PU composites. Nevertheless, the reinforcing effect of LCP is more pronounced in CF/PA-PU composite compared to Gr. The addition of 1 wt.% LCP to the CF/PA-PU composite resulted in a tensile strength of 91.37 MPa, which is 16.98% higher than that of the CF/PA-PU composite (78.11 MPa). The maximum improvement in tensile strength was observed in the 5%LCP/CF/PA-PU composite, with an increase of 26.85%. The enhancement of the LCP in CF/PA-PU composites is mainly attributed to its ‘in situ’ composite enhancement effect, i.e., during the processing of composites, a microfiber structure is formed by the liquid crystal rod molecules in the blend matrix [24]. Furthermore, the hydrogen bonding between the aromatic copolyester LCP and the PA-PU copolymer promotes stability and compatibility within blends [29]. A small amount of LCP can provide reinforcement effects equivalent to those achieved by many fibers while maintaining or even improving the original processing properties of the matrix. The addition of LCP results in a lower melt viscosity, which promotes the uniform dispersion of carbon fibers in composites and facilitates the formation of a favorable interface with the matrix, which is demonstrated in Figure 3. It is worth noting that the use of LCP slightly increased the elongation at break for CF/PA-PU composites. Excessive LCP caused self-aggregation and poor compatibility, and thus the tensile strength of the 7%LCP/CF/PA-PU composite decreases slightly, but it is still better than that of the CF/PA-PU composite. Figure 2b shows that the Young’s modulus of the LCP/CF/PA-PU composites increases monotonously with increasing LCP content, from 1932.39 MPa for CF/PA-PU to 2204.60 MPa for 7%LCP/CF/PA-PU, due to the excellent mechanical properties of the LCP itself.

### 3.2. Tribological Properties

To investigate the effects of LCP on the tribological properties of CF/PA-PU composites, friction and wear tests were conducted and the results were compared with those obtained by commonly-used rigid lubricant Gr. At the beginning of the sliding friction and wear test, all specimens experienced a rapid increase in the coefficient of friction (COF), which was related to an increase in the actual contact area between the specimen test surface and the friction pair. The COF tends to be stable after a transfer film is formed on the sample and the steel plate surface, and then the COF reported here is calculated from average values during the stable friction process. Meanwhile, the scar width (the arc chord length of the cross-section of the damaged trace left by the wear surface of the sample after testing) and the wear rate were used to characterize the wear of the sample. The specific wear rate was calculated using the equation:

$$\mathrm{wear}\;\mathrm{rate}=\frac{\Delta V}{Ld}$$

where Δ*V* is the wear volume calculated from the ratio of wear mass to specimen density, *L* is the normal load, and *d* is the sliding distance.

**Figure 4:**
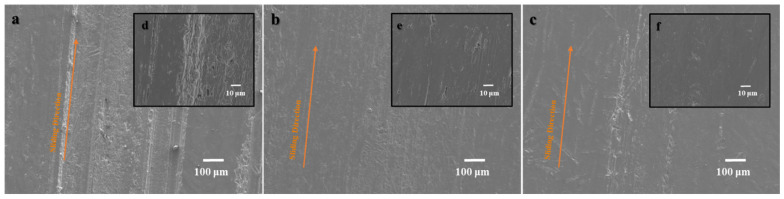
SEM images of the worn surfaces of the specimens: (**a**) CF/PA-PU, (**b**) 3%Gr/CF/PA-PU, and (**c**) 5%LCP/CF/PA-PU. The insets in (**a**–**c**) are taken at high magnification.

During the sliding process, the heat and external force caused by friction soften the polymer matrix. As depicted in Figure 4a,d, plucked and ploughed marks were found on the wear surface of the CF/PA-PU composite, indicating an adhesive and plough wear mechanism. Nevertheless, the carbon fibers exposed to the surface during the friction process act as a solid lubricant to mitigate this phenomenon. Incorporating Gr or LCP into CF/PA-PU composites enhanced their mechanical properties, enabling them to effectively resist the plastic deformation of the matrix, which could be inferred from the relatively smooth wear surfaces of the 3%Gr/CF/PA-PU and 5% LCP/CF/PA-PU composites in Figure 4.

**Figure 5:**
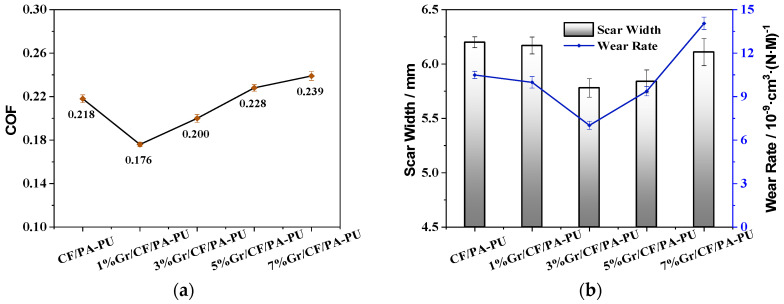
(**a**) Coefficient of friction, (**b**) scar width, and wear rate of Gr/CF/PA-PU composites with different contents of Gr.

**Figure 6:**
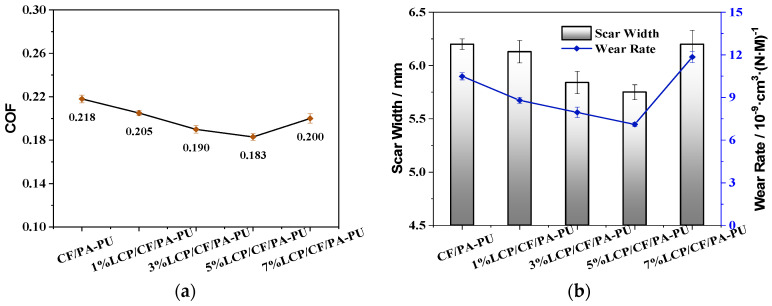
(**a**) Coefficient of friction, (**b**) scar width, and wear rate of LCP/CF/PA-PU composites with different contents of LCP.

Figure 5a and Figure 6a illustrate the COF of CF/PA-PU composites with Gr and LCP, respectively. As the filler content increases, the COF, scar width, and wear rate of both the Gr/CF/PA-PU and the LCP/CF/PA-PU composites decrease first and then increase.

Figure 5a demonstrates that the introduction of 1 wt.% Gr into the CF/PA-PU composite results in a decrease of 19.27% in COF compared to the CF/PA-PU composite, which had a COF of 0.218. As shown in Figure 5b, the scar width and wear rate of 3%Gr/CF/PA-PU reach the lowest values of 5.78 mm and 7.02 × 10^−9^·cm^3^·(N·M)^−1^, which are 6.77% and 33.08% lower than those of the CF/PA-PU composite, respectively. Gr, as a solid lubricant, can be released from the matrix during the sliding process. Then, Gr was exposed and attached to the wear surface to form a graphite crystal transfer film, resulting in lower COF, scar width, and wear rate for the composites [16]. The aggregation and poor dispersion of the Gr can cause stress concentration and defects in the composite, such as microcracks and voids, which are observed in Figure 4e. The higher content of graphite has further exacerbated this phenomenon, making it easier to pull out the fillers from the matrix. As a result, there is an increase in COF, scar width, and wear rate.

Although the rill-like wrinkles mainly caused by adhesive wear can still be observed in Figure 4c,f, the friction conditions of LCP/CF/PA-PU wear surfaces have been significantly improved compared to CF/PA-PU. The extrusion deformation of the carbon fiber and the filling effect of the wear debris cause the wear surface to be compact and smooth, thereby improving the friction condition. A stable transfer film is formed on the counterpart surface, which is a key mechanism for achieving low friction and wear [11]. As shown in Figure 6a,b, the COF, scar width, and wear rate of LCP/CF/PA-PU decrease monotonically with increasing LCP content until 7 wt.% LCP, which is consistent with their mechanical properties. The CF/PA-PU containing 5 wt.% LCP shows a 16.06% decrease in COF (0.183), and exhibits the lowest scar width (5.75 mm) and wear rate (7.11 × 10^−9^·cm^3^·(N·M)^−1^), about 7.26% and 32.22% lower than those of the CF/PA-PU composite. The excellent tribological properties of the LCP/CF/PA-PU composite were attributed to a synergistic effect between the superior self-enhancement properties of LCP and probably a rolling effect induced by chopped CF [30]. LCP could form highly oriented crystalline structures, i.e., liquid crystalline microfibrils, when subjected to shear flow above its melting point. LCP acted as a reinforcing agent in composites, and then the planar structure formed by the conjugation of two benzene rings in LCP facilitates the orderly arrangement of molecules to form shear planes conducive to lubrication [25]. In addition, LCP allowed the composites to have a low viscosity during processing as discussed in the next section, which promotes good impregnation between the carbon fiber and the polymer matrix. The good adhesion of LCP to chopped carbon fiber is beneficial for the role of carbon fiber as a skeleton to carry stress, which can significantly reduce the plastic deformation and cavitated areas of composites. An increase in LCP content beyond 5% results in an upward trend of COF, scar width, and wear rate. Nevertheless, the COF of the 7%LCP/CF/PA-PU composite is still lower than that of the CF/PA-PU composite. Excessive LCF worsens its compatibility, making it easier for both LCP and CF to be pulled out from the matrix.

### 3.3. Processing Properties

**Figure 7:**
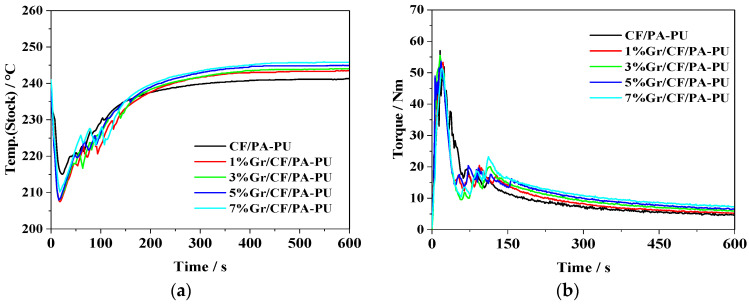
(**a**) Temperature–time and (**b**) torque–time curves of CF/PA-PU composites with different contents of Gr.

**Figure 8:**
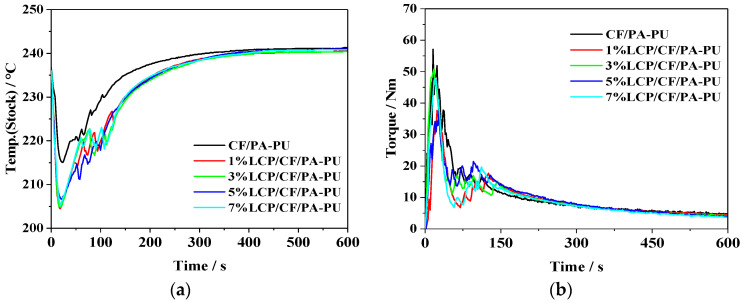
(**a**) Temperature–time and (**b**) torque–time curves of CF/PA-PU composites with different contents of LCP.

**Figure 9:**
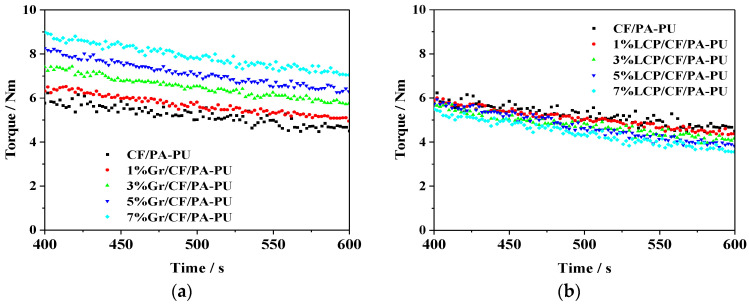
Torque–time curve (400–600 s) of CF/PA-PU composites with different contents of (**a**) Gr and (**b**) LCP respectively.

In industrial production, this is the key to solving the problem of processing properties for the development of new materials. Thus, it is necessary to further investigate the effect on the processing properties of CF/PA-PU composites. A torque rheometer is employed to simulate actual production conditions and detect the changes in torque and temperature over time. After the end of the feeding stage, the processing temperature eventually tends to converge near the set temperature, while the torque reaches equilibrium with the complete melting and uniform mixing of the composite. The value of the torque directly reflects the apparent viscosity of the composites to a certain extent, and the energy consumption represents the energy consumed per unit mass during the entire processing stage, which is given by the test system. The temperature–time curve and torque–time curve of CF/PA-PU composites are given in Figure 7, Figure 8 and Figure 9. The detailed data are summarized in Table 2.

As shown in Figure 7b and Figure 9a, the addition of rigid fillers, such as Gr, to the composites increases their melt viscosity in proportion to the filler content, as evidenced by the increase in equilibrium torque with increasing Gr content. Furthermore, it can be observed in Figure 7a that an increase in Gr content causes the equilibrium temperature of the composite melt to be higher than its set temperature. This indicates that there is intensified friction within the chamber due to the increased Gr content, which could potentially lead to composite degradation, high energy consumption during processing, and equipment abrasion.

Composites containing LCP exhibit processing properties that offer promising advantages compared to rigid fillers such as Gr. When LCP melts in the liquid crystalline state, intermolecular entanglement is reduced, intermolecular friction is low, and the molecules are prone to spontaneous orientation. These typical characteristics of LCP make it useful as a ‘plasticizer’ to reduce the entanglement of molecular chains and reduce the melt viscosity of the compounding system [26,27,28]. Figure 8b and Figure 9b show that the equilibrium torque decreases with increasing LCP content. When the LCP content is 7 wt.%, the lowest equilibrium torque is only 3.64 Nm, which is reduced by 24.01% compared to the CF/PA-PU composite. In addition, the trend of energy consumption is consistent with that of the equilibrium torque, from 0.472 KNm·g^−1^ for CF/PA-PU to 0.401 KNm·g^−1^ for 7%LCP/CF/PA-PU. The lower melt viscosity is beneficial for the uniform dispersion of carbon fibers and the formation of liquid crystal microfibrils, which could promote the interfacial bonding between the polymer matrix and carbon fibers [30], thus further improving the mechanical and tribological properties of LCP/CF/PA-PU composites. Furthermore, Figure 8a indicates that the equilibrium temperature of the composite melts hardly deviates from the set temperature with the addition of LCP, which could ensure the stability of the composites throughout the production processes.

### 3.4. Thermal Properties

**Figure 10:**
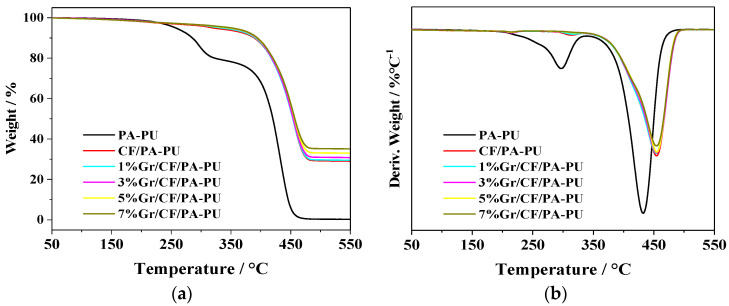
(**a**) TG and (**b**) DTG curves of CF/PA-PU composites with different contents of Gr.

**Figure 11:**
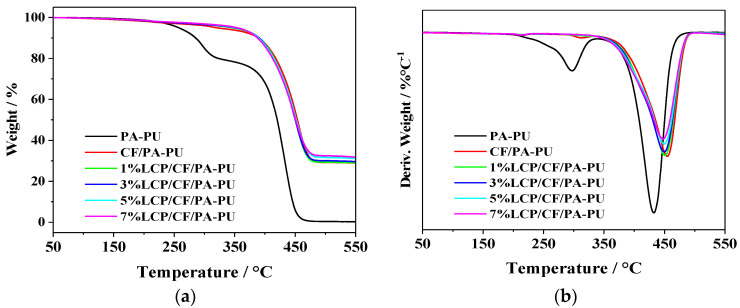
(**a**) TG and (**b**) DTG curves of CF/PA-PU composites with different contents of LCP.

Thermal stability is the key factor that directly affects the processing condition and application of composites. Figure 10 and Figure 11 show the TG and DTG curves of the PA-PU copolymer, CF/PA-PU composite, and CF/PA-PU composites with different contents of Gr or LCP. The relative values are given in Table 3. It can be perceived that the addition of carbon fiber has greatly improved the thermal stability of the PA-PU copolymer, which increases its initial thermal decomposition temperature by about 20%, and then the maximum decomposition rate and the mass loss of the first stage drop considerably, which indicates that the process of caprolactam unblocking decomposition is mitigated and delayed. The presence of Gr or LCP will further increase the initial thermal decomposition temperature of CF/PA-PU composites, and the LCP is more effective in improving the thermal stability of CF/PA-PU composites. After adding Gr (1 wt.%) or LCP (1 wt.%), the initial decomposition temperatures of the composites increase to 333.54 °C and 346.05 °C, respectively, and continue to increase with the increase in Gr or LCP content separately. The residual mass was positively correlated with the amount of Gr or LCP in the composites, which indicates the good thermal stability of Gr and LCP.

In the presence of LCP, the first stage of decomposition disappears completely. For the second stage of decomposition, Gr and LCP hardly change the temperature of the maximum decomposition rate, but the maximum decomposition rate gradually decreased with the increase in LCP content. This is supported by the superior thermal properties of LCP, which imparts improved thermal stability to the LCP/CF/PA-PU composites.

## 4. Conclusions

The addition of Gr to CF/PA-PU composites could enhance the tribological properties without compromising the mechanical properties. For instance, in the 3%Gr/CF/PA-PU composite, the wear rate decreased by 33.08% compared to CF/PA-PU, and the tensile strength hardly changed. However, the addition of 3 wt.% Gr resulted in a 21.71% increase in equilibrium torque and an elevated processing temperature.

The LCP/CF/PA-PU composite benefits from the self-reinforcement effects and good flowability of LCP. By incorporating 5 wt.% LCP, the tensile strength of the LCP/CF/PA-PU composite was increased by 26.85%, to reach 99.08 MPa, while reducing the COF by 16.06% and the wear rate by 32.22%, in comparison with those of CF/PA-PU. Furthermore, these improvements were achieved while maintaining the desirable processing properties, as evidenced by a lower equilibrium torque than that of CF/PA-PU. In addition, the thermal stability of LCP/CF/PA-PU composites was also significantly improved. The initial decomposition temperature of 5%LCP/CF/PA-PU increased by 14.19% compared to that of CF/PA-PU and effectively slowed down its maximum decomposition rate.

Our work demonstrates that LCP is an effective filler to improve the tribological properties of carbon-fiber-reinforced PA-PU composites. Moreover, it has great potential to further improve the mechanical properties and overcome the processing difficulties associated with CF/PA-PU composites.

## Figures and Tables

**Table 1:** Formulations of CF/PA-PU composites with different contents of Gr or LCP.

Samples	PA-PU (wt.%)	CF (wt.%)	Gr (wt.%)	LCP (wt.%)
CF/PA-PU	70.0	30.0	0	0
1%Gr/CF/PA-PU	69.0	30.0	1.0	0
3%Gr/CF/PA-PU	67.0	30.0	3.0	0
5%Gr/CF/PA-PU	65.0	30.0	5.0	0
7%Gr/CF/PA-PU	63.0	30.0	7.0	0
1%LCP/CF/PA-PU	69.0	30.0	0	1.0
3%LCP/CF/PA-PU	67.0	30.0	0	3.0
5%LCP/CF/PA-PU	65.0	30.0	0	5.0
7%LCP/CF/PA-PU	63.0	30.0	0	7.0

**Table 2:** Processing properties of CF/PA-PU composites with different contents of Gr or LCP.

Samples	Equilibrium Torque /Nm	Equilibrium Temperature /°C	Energy Consumption /KNm·g^−1^
CF/PA-PU	4.79 ± 0.10	241.39 ± 0.25	0.472 ± 0.004
1%Gr/CF/PA-PU	5.10 ± 0.03	243.46 ± 0.32	0.505 ± 0.007
3%Gr/CF/PA-PU	5.83 ± 0.09	244.20 ± 0.43	0.524 ± 0.004
5%Gr/CF/PA-PU	6.41 ± 0.10	245.41 ± 0.24	0.564 ± 0.008
7%Gr/CF/PA-PU	7.22 ± 0.09	246.30 ± 0.33	0.570 ± 0.012
1%LCP/CF/PA-PU	4.43 ± 0.11	240.43 ± 0.15	0.469 ± 0.004
3%LCP/CF/PA-PU	4.16 ± 0.04	240.27 ± 0.52	0.452 ± 0.002
5%LCP/CF/PA-PU	3.91 ± 0.05	240.95 ± 0.24	0.436 ± 0.003
7%LCP/CF/PA-PU	3.64 ± 0.11	240.82 ± 0.33	0.401 ± 0.009

**Table 3:** Thermal properties of CF/PA-PU composites with different contents of Gr and LCP.

Samples	Initial Decomposition Temperature T_5%_/°C	Temperature of the Maximum Decomposition Rate/°C		Mass Loss/%		Residual Mass/%
		Stage 1	Stage 2	Stage 1	Stage 2	
PA-PU	257.29	296.33	433.89	21.10	78.72	0.18
CF/PA-PU	308.96	296.48	454.54	5.62	65.30	29.08
1%Gr/CF/PA-PU	333.54	316.84	454.52	5.05	65.45	29.50
3%Gr/CF/PA-PU	345.04	-	455.12	-	69.20	30.80
5%Gr/CF/PA-PU	345.84	-	455.52	-	67.03	32.97
7%Gr/CF/PA-PU	349.84	-	455.63	-	64.90	35.10
1%LCP/CF/PA-PU	346.05	-	451.39	-	70.78	29.22
3%LCP/CF/PA-PU	349.77	-	450.15	-	70.08	29.92
5%LCP/CF/PA-PU	352.79	-	449.93	-	68.46	31.54
7%LCP/CF/PA-PU	354.30	-	448.03	-	67.65	32.35

## Data Availability

The data presented in this study are available on request from the corresponding author.
